# Supplementary material for: Characterizing cell-type spatial relationships across length scales in spatially resolved omics data
Source: Nat Commun. 2025 Jan 3;16:350. doi: 10.1038/s41467-024-55700-1 (PMC11699133; doi:10.1038/s41467-024-55700-1)
Supplement: Supplementary file 3 — Supplementary Software 1 [file 41467_2024_55700_MOESM3_ESM.zip › CRAWDAD/plots/supplementary/S7_cluster_corr_heatmap_PBVN_vs_FSLD.pdf]

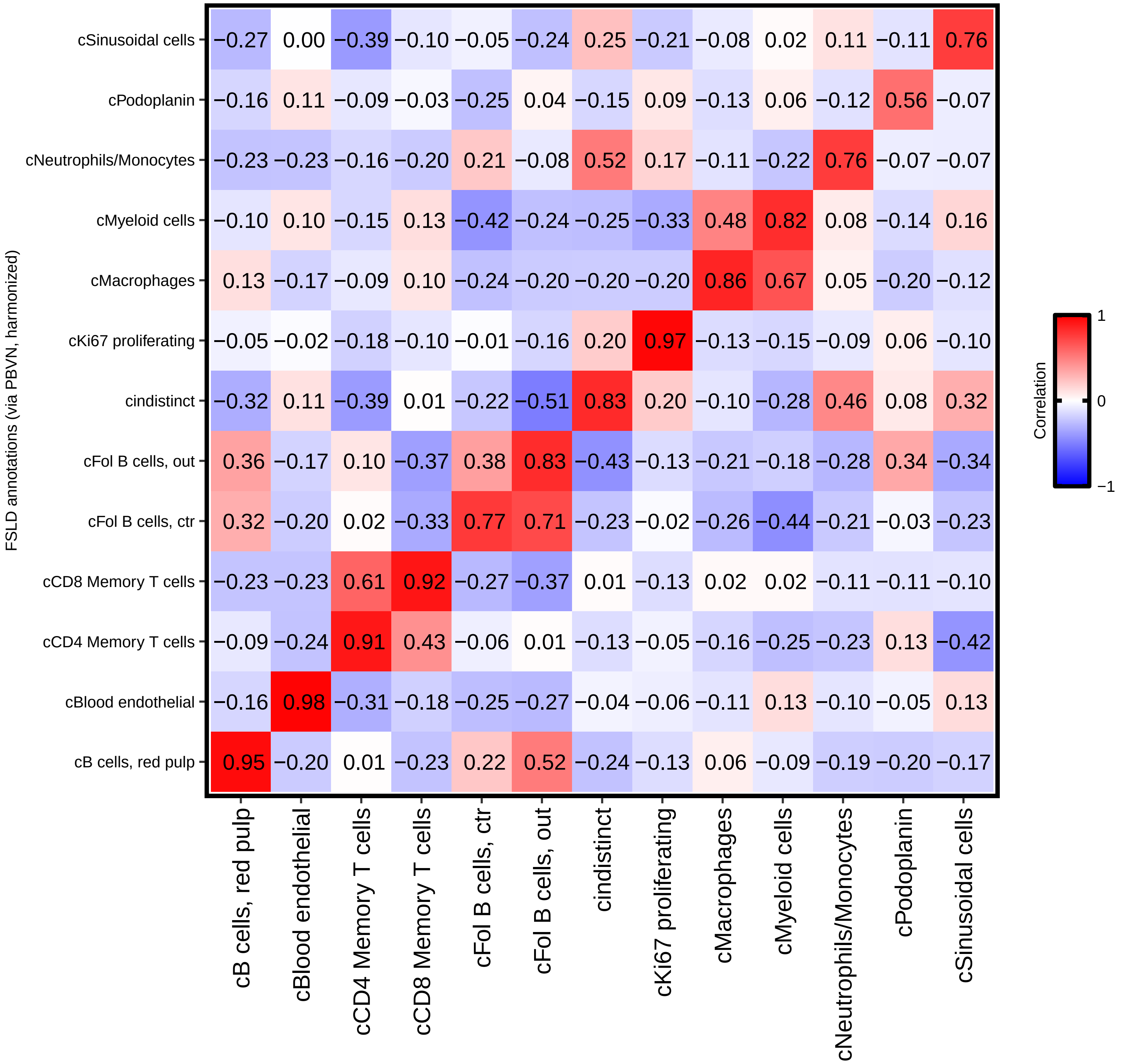
cSinusoidal cells
cPodoplanin
cNeutrophils/Monocytes
cMyeloid cells
cMacrophages
cKi67 proliferating
cindistinct
cFol B cells, out
cFol B cells, ctr
cCD8 Memory T cells
cCD4 Memory T cells
cBlood endothelial
cB cells, red pulp
FSLD annotations (via PBVN, harmonized)
−0.27 0.00 −0.39 −0.10 −0.05 −0.24 0.25 −0.21 −0.08 0.02 0.11 −0.11 0.76
−0.16 0.11 −0.09 −0.03 −0.25 0.04 −0.15 0.09 −0.13 0.06 −0.12 0.56 −0.07
−0.23 −0.23 −0.16 −0.20 0.21 −0.08 0.52 0.17 −0.11 −0.22 0.76 −0.07 −0.07
−0.10 0.10 −0.15 0.13 −0.42 −0.24 −0.25 −0.33 0.48 0.82 0.08 −0.14 0.16
0.13 −0.17 −0.09 0.10 −0.24 −0.20 −0.20 −0.20 0.86 0.67 0.05 −0.20 −0.12
−0.05 −0.02 −0.18 −0.10 −0.01 −0.16 0.20 0.97 −0.13 −0.15 −0.09 0.06 −0.10
−0.32 0.11 −0.39 0.01 −0.22 −0.51 0.83 0.20 −0.10 −0.28 0.46 0.08 0.32
0.36 −0.17 0.10 −0.37 0.38 0.83 −0.43 −0.13 −0.21 −0.18 −0.28 0.34 −0.34
0.32 −0.20 0.02 −0.33 0.77 0.71 −0.23 −0.02 −0.26 −0.44 −0.21 −0.03 −0.23
−0.23 −0.23 0.61 0.92 −0.27 −0.37 0.01 −0.13 0.02 0.02 −0.11 −0.11 −0.10
−0.09 −0.24 0.91 0.43 −0.06 0.01 −0.13 −0.05 −0.16 −0.25 −0.23 0.13 −0.42
−0.16 0.98 −0.31 −0.18 −0.25 −0.27 −0.04 −0.06 −0.11 0.13 −0.10 −0.05 0.13
0.95 −0.20 0.01 −0.23 0.22 0.52 −0.24 −0.13 0.06 −0.09 −0.19 −0.20 −0.17
cB cells, red pulp
cBlood endothelial
cCD4 Memory T cells
cCD8 Memory T cells
cFol B cells, ctr
cFol B cells, out
cindistinct
cKi67 proliferating
cMacrophages
cMyeloid cells
cNeutrophils/Monocytes
cPodoplanin
cSinusoidal cells
PBVN annotations (via PKHL)
Correlation
1
0
−1
